# Harnessing the Potential of Human iPSC‐derived Microglia‐like Cells as Antigen Presenting Cells for MAIT Cells to Study AD Pathology

**DOI:** 10.1002/alz.087699

**Published:** 2025-01-03

**Authors:** Reham Afify, Randy R Brutkiewicz

**Affiliations:** ^1^ Stark Neurosciences Research Institute, Indiana University School of Medicine, Indianapolis, IN USA

## Abstract

**Background:**

Microglia are dominant immune cells residing in the brain that regulate brain homeostasis and T‐cell responses. An important immune function of microglia involves presenting microbial antigens to mucosal‐associated invariant T (MAIT) cells; MAIT cells recognize microbial vitamin B‐derived metabolites presented by the MHC class I‐like molecule, MR1. Our recent findings highlighted a detrimental role for the MR1/MAIT cell axis in Alzheimer’s disease (AD) using the 5XFAD mouse model. Here, our work is focused on how the MR1/MAIT cell innate immune axis in humans contributes to AD, using iPSC‐derived human microglia‐like cells (iMG) to provide potential mechanistic insights underlying the MR1/MAIT cell axis in AD. As an initial evaluation, we have assessed the activation of iMG and their potential as functional antigen presenting cells (APC) to MAIT cells when exposed to a microbe that can provide a MR1‐presented, MAIT cell‐specific antigen, such as from *E. coli*.

**Methods:**

Initially, we will generate iMG from healthy donors that will be incubated with *E. coli* for the assessment TNF‐α levels using ELISA as a readout of microglial activation. Subsequently, coculture experiments with human MAIT cells and *E. coli*‐stimulated iMG will provide an initial evaluation of microglial MR1‐dependent MAIT cell activation via MR1‐dependent antigen presentation, monitored by MAIT cell production of various cytokines measured by ELISA.

**Results:**

We expect that iMG will produce increased levels of TNF‐α in response to *E. coli* compared to the control group. Similarly, we anticipate elevated cytokine levels secreted by MAIT cells via an MR1‐dependent pathway.

**Conclusions:**

These experiments will allow us to apply our human iPSC‐based model to studying human microglia as APC and pave the way for a broader understanding of the contributions of the MR1/MAIT cell axis in AD. To our knowledge, our proposed study will be the first of its kind to utilize iMG as APC for human MAIT cells.

